# Evaluation of the clinical and sociodemographic features of patients with vitiligo from the central region of Romania

**DOI:** 10.3389/fmed.2025.1544184

**Published:** 2025-03-06

**Authors:** Laszlo Fekete, Gyula Laszlo Fekete, Laszlo Barna Iantovics, Júlia Edit Fekete, Vladimir Bacârea

**Affiliations:** ^1^Doctoral School, George Emil Palade University of Medicine, Pharmacy, Science and Technology of Targu Mures, Târgu Mureş, Romania; ^2^CMI Dermamed Private Medical Office, Târgu Mureş, Romania; ^3^Department of Dermatology, George Emil Palade University of Medicine, Pharmacy, Science and Technology of Targu Mures, Târgu Mureş, Romania; ^4^Department of Electrical Engineering and Information Technology, George Emil Palade University of Medicine, Pharmacy, Science and Technology of Targu Mures, Târgu Mureş, Romania; ^5^National Institute of Public Health, Regional Center for Public Health, Târgu Mureş, Romania; ^6^Department of Research Methodology, George Emil Palade University of Medicine, Pharmacy, Science and Technology of Targu Mures, Târgu Mureş, Romania

**Keywords:** vitiligo, sociodemographic features, clinical features, cohort, pigmentation disorder, autoimmune disease

## Abstract

**Background:**

In this study, we aimed to investigate and analyze the clinical and sociodemographic features and possible correlation with factors associated with the development of vitiligo in a cohort of patients suffering from this disease in the central region of Romania.

**Methods:**

Patients diagnosed with vitiligo from private outpatient clinics in the region and from the outpatient clinic of the Dermatology Clinic in Târgu Mureş participated in the study. The study period was between March 2021 and March 2022. Both sets of patients adhered to the same specified inclusion and exclusion criteria. Included just patients who received a complete dermatological clinical examination. They were asked by experienced physicians about epidemiological and clinical data of the disease, using the questions about vitiligo from the validated questionnaire edited by the Vitiligo Research Foundation from the United States of America. The patients who were given incomplete responses were excluded. This questionnaire contains 30 questions with multiple answers, about the patients with vitiligo, divided into seven subgroups as follows: group 1. Origins (demographic data), 2. History of vitiligo, 3. Vitiligo description, 4. Vitiligo treatments, 5. Skin condition, 6. Other conditions (comorbidities), 7. Impact (cost of treatments). Our study consisted of 114 patients, all of whom were Caucasians with Fitzpatrick skin types ranging from I–III.

**Results:**

We have analyzed the found data and compared the result with the data found in the literature. Most of the clinical and epidemiological characteristics of vitiligo in our patients were similar to those in other studies. A few of the characteristics linked to the possible appearance of the disease were present in higher percentages like the presence of the disease in the family, lighter color of the eyes, gray colored hair, the presence of the halo naevus, the predisposition to sunburn, the skin trauma as starting cause and the presence of increased level of thyroid disease.

**Conclusion:**

Based on our results, we can conclude a profile of a potential patient who can develop vitiligo. To our knowledge, this study is the first of its kind from our country, however, our inferences remain limited by the single center, a relatively small sample size, recall bias, and a self-decided classification of some clinical aspects, which are potential limitations of this study.

## 1 Introduction

Vitiligo is a chronic autoimmune pigmentary skin disease. It is estimated to affect between 0.5 and 4% of the global population with a documented prevalence of approximately 1% of the world population (1, 2). It is an acquired noncontagious disease characterized by progressive patchy loss of pigmentation of the skin, overlying hair, and mucous membranes (3). The highest incidence has been reported in India, followed by Mexico and Japan (4, 5). The disease can start at any age; appearing rarely in childhood and the elderly, generally the highest incidence is among young adults. The physiopathology of the disease is complex and still unclear, but the basic mechanism is the progressive loss of functional melanocytes and/or their products due to different causes (6). Vitiligo results from many factors. Several hypotheses claim that factors such as stress, environmental compounds, skin trauma, autoimmunity, microorganisms, genetic predisposition, altered cellular environment, and affected melanocyte functions can contribute to the development of vitiligo (7–10). Others consider vitiligo a systemic disorder in which a spectrum of many disorders with different etiologies and pathogeneses, can cause the same issue, the loss of melanocytes (11). There are several clinical types of vitiligo with possible different complex mechanisms of appearance (12). In this study, we aimed to investigate and analyze the clinical and sociodemographic features and possible correlation with factors associated with the development of vitiligo in a cohort of patients suffering from this disease in the central region of Romania. To our knowledge, this is the first study about this issue in our country.

## 2 Materials and methods

Patients diagnosed with vitiligo from private outpatient offices in the region and the outpatient department of the Dermatology Clinic in Târgu Mureş participated in the study. The study period was between March 2021 and March 2022. All patients adhered to the same specified inclusion and exclusion criteria. Inclusion criteria encompassed patients over 18 years of age, diagnosed with vitiligo of any form, and who signed the informed consent before data collection. All the patients received a complete dermatological clinical examination performed by experienced dermatologists who were involved voluntarily. At the same time, the patients were asked about epidemiological and clinical data of the disease, using the questions about vitiligo from the questionnaire edited by the Vitiligo Research Foundation from the United States of America (13). This questionnaire contains 30 questions with multiple answers, about the patients with vitiligo, divided into seven subgroups as follows: group 1. Origins (demographic data) 3 questions, 2. History of vitiligo 6 questions, 3. Vitiligo description 3 questions, 4. Vitiligo treatments 7 questions, 5. Skin condition 4 questions, 6. Other conditions (comorbidities) 4 questions, 7. Impact (cost of treatments) 1 question. Patients’ data with incomplete responses were excluded. The enrolled participants have given informed consent, outlining the confidentiality of their data. The data collected from the clinical examination and the questionnaires were recorded in a database. The data has been encoded in compliance with the General Data Protection Regulation (GDPR).

The established minimal sample size after the exclusion criteria application and removing the inadequate data (like missing responses to important questions) was 100, which is usual in such studies considering also the fact that vitiligo is very rare.

The normality test was performed for the age of the patients. There were applied two numerical normality tests, namely Lilliefors, and Shapiro–Wilks. The actual sample size permitted the application of both tests. Must be noticed that the Shapiro–Wilks test works very well even for very low sample sizes (lower than 30), and has a better power compared to other tests like Kolmogorov Smirnov, Lilliefors, and Anderson Darling.

## 3 Results

### 3.1 Origins (demographic data)

After applying the exclusion criteria to the collected data by removing some patients’ data, for study remained 114 patients, all of whom were Caucasians with Fitzpatrick skin types ranging from I–III. Out of the total patients, 56 (49.12%, 95% CI 39.81–58.44%) patients were male and 58 (50.88%,95% CI 41.56–60.19%) patients were female. Statistical comparison of percentages shows no statistical difference (*p* = 0.7909). The average age was 49, with 95% CI [45.8, 52.3], a median age of 49, a range of 59, with a minimum of 19, and a maximum of 78 years (Table 1).

**TABLE 1:** Distribution of cases by age.

	Statistic	Std. error
Mean	49.05	1.641
95% confidence Interval for mean	45.8–52.3	
5% trimmed mean	49.12	
Median	49	
Variance	307	
Std. deviation	17.52	
Minimum	19	
Maximum	78	
Range	59	
Interquartile range	34	
Skewness	−0.01	0.226
Kurtosis	−1.286	0.449

Descriptive statistics.

**FIGURE 1:**
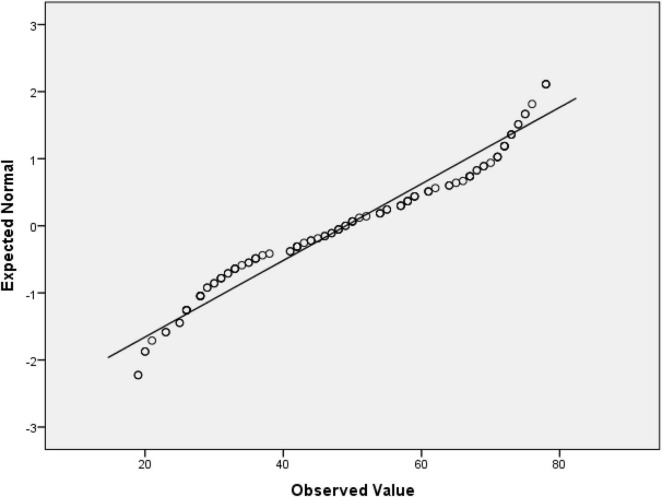
Q-Q plot of age.

The result presented in Table 2 shows that the age is not normally distributed. Figure 1 visually validates the failure to pass the normality assumption.

**TABLE 2:** Testing the age normality.

Lilliefors		Shapiro–Wilk	
**Statistics**	***P***	**Statistic**	***P***
0.096	0.011	0.944	0

Lilliefors tests of normality for age.

Considering the age of the studied vitiligo patients for appropriate analysis, consulting our epidemiologist coautors, as follows, resulting: 1. Age group 18–40 years: 40 patients, (35.1%), of which 22 (19.3%) were male and 18 (15.8%) were female. 2. Age group 41–60 years: 37 patients (32.5%), of which 18 (15.8%) were male and 19 (16.7%) were female. 3. Age group > 61 years: 37 patients (32.5%), of which 16 (14%) were male and 21 (18.4%) were female.

**FIGURE 2:**
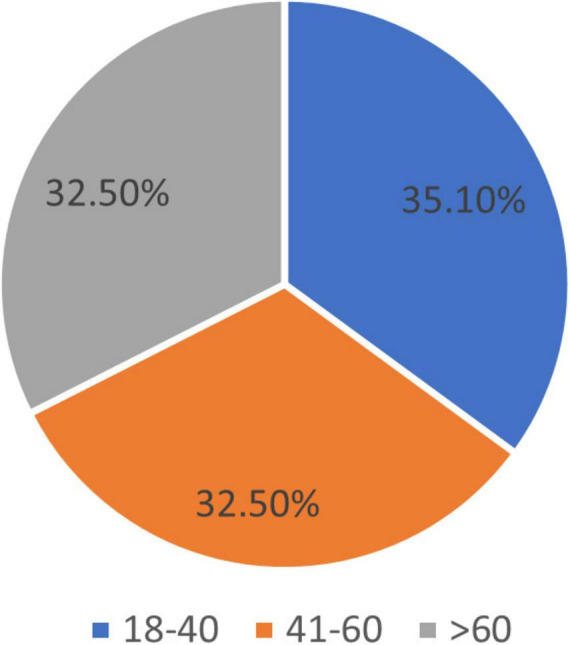
Age groups.

**FIGURE 3:**
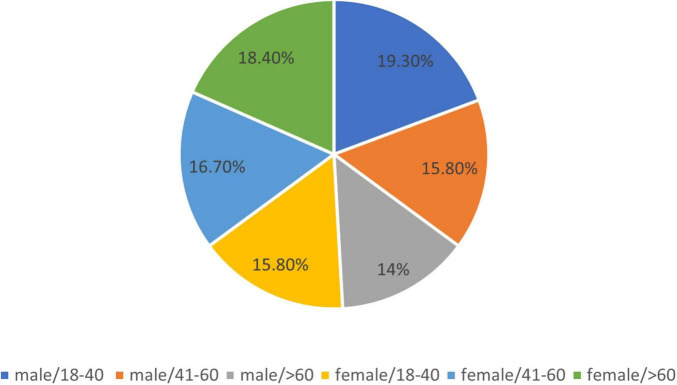
Age and gender distribution.

The results are shown in Figures 2, 3.

Of the patients, 76 (66.67%) of them reside in urban areas, while 38 (33.33%) are from rural areas.

**FIGURE 4:**
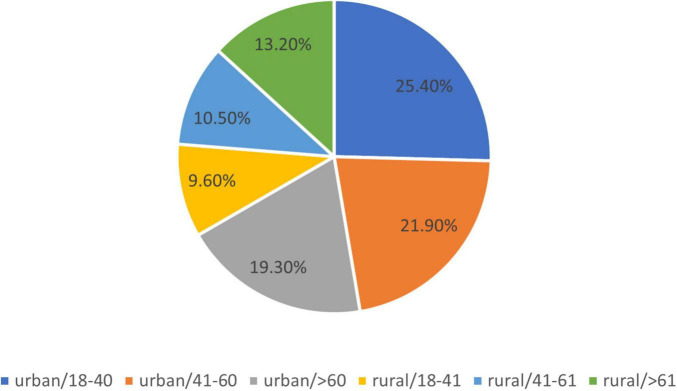
Residents by age of the group.

The results regarding the residents of the patients by age group were as follows (Figure 4):

1. Urban area: 76 patients of which in the 18–40 age group, there were 29 patients (25.4%), in the 40–60 age group there were 25 patients (21.9%), and in the age group > 60 years were 22 patients (19.3%).
2. Rural area: 38 patients, of which there were 11 patients (9.6%) in the 18–40 age group, 12 patients (10.5%) in the 40–60 age group, and age > 60 years were 15 patients (13.2%).

**FIGURE 5:**
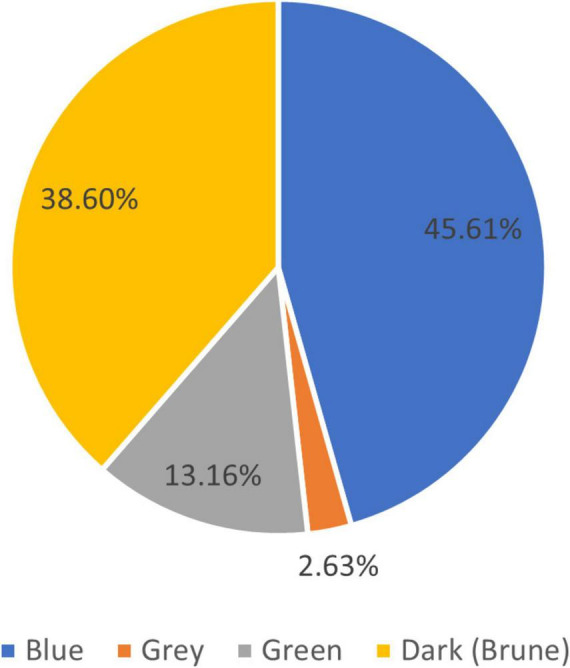
Eye color distribution.

The results of these studies show that from the total number of patients, 5 have completed primary education, 73 secondary education, and 36 higher education. In terms of marital status, 77 (67.54%) were married and 37 (32.46%) patients were unmarried. The last question from this subgroup was about the color of the eyes. Looking at eye color, the results are mentioned in Figure 5. Of the patients studied, 70 (61.4%) have light-colored eyes.

### 3.2 History of vitiligo

Regarding the question about the age of the first signs of vitiligo, for easier analysis, we decided to group the answers as follows: group 1. Less than five years, group 2. between 5 and 10 years and group 3 more than 10 years of appearance. In the first group, there were 27 (23.7%) patients, in group 24 (21.05%) patients, and in the group 3 (55.25%) patients.

**FIGURE 6:**
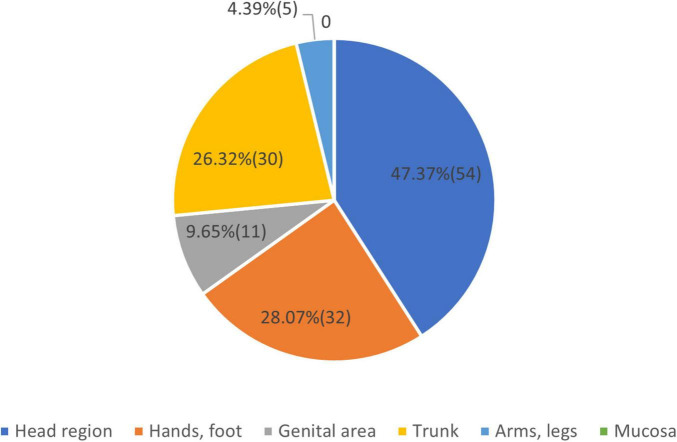
Vitiligo first sign appeared on.

About the first location of the disease, the results are mentioned in Figure 6.

**FIGURE 7:**
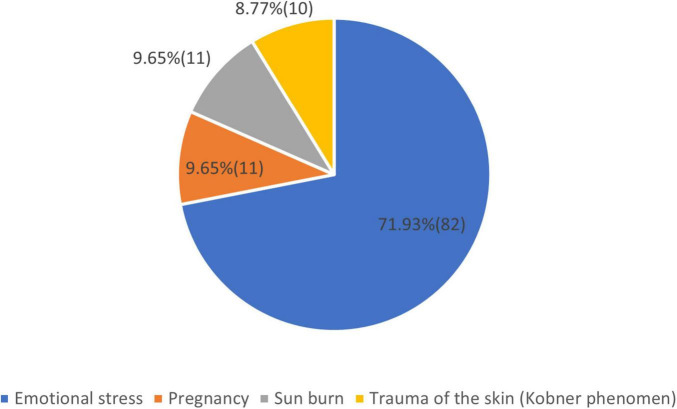
Possible causes of the appearance of vitiligo.

We note that in some people the first sign of the disease appeared in more than one place. We have chosen the larger surfaced lesion as the first clinical sign. As the condition evolved other locations appeared, which changed the allocation of the clinical form. The question regarding the possible causes of the appearance of the disease is summarized in Figure 7.

Regarding the progression of vitiligo during the last three months, 82 (71.9%) of the patients answered that it was stable, and 32 (28.1%) answered that was active. Regarding the progress of the disease earlier 76 (66.66%) patients declared a slow progressive spreading over years and 38 (33.33%) patients presented quick, short bursts then limited spreading. Regarding the question about noticing redness or itch before the disease appears 82 (72%) answered yes and 32 (28%) answered no.

### 3.3 Vitiligo description

The answers to the question describing skin color were updated by us to the Fitzpatrick skin type scale. Results show that 12 (10.5%) patients were in type I, 82 (72%) in type II, and 20 (17.5%) were in type III according to the classification.

For the other two questions regarding the clinical form and affected area, we decided to group them according to localization on visible areas: facial, hands, and foot region, named Group = 0, containing 47 (41.2%) patients, and localization on invisible areas: trunk, mucosal, genital form, and other forms that appear especially on the body named Group = 1, containing 67(58.8%) patients. The affected surface is a criterion for the severity of the disease, which is why we grouped them as skin involvement below 10% of the skin, minor form: Group 1 = 54 (47.4%) patients, skin involvement between 10 and 25% of the skin, moderate form: Group 2 = 46 (40.4%) patients and skin involvement over 25% of the skin, severe form: Group 3 = including 14 (12.2%) patients.

### 3.4 Vitiligo treatments

**FIGURE 8:**
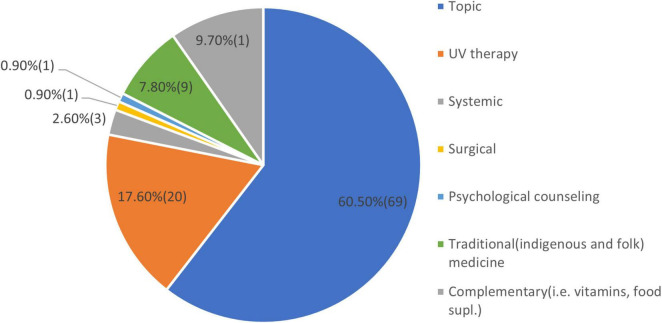
Type of treatments used.

Analyzing the seven questions about this topic we got the following results. The type of treatment patients used is shown in Figure 8.

At the time of the latest treatment, the disease of the patients was active in 72 (63.2%) patients and was stable in 28 (36.8%) of the patients. As a result of the latest treatment the disease continued with new patches at 22 (19.3%) patients and stopped with remaining patches at 92 (80.7%) of the patients. The latest treatment duration of the patients had an average period of 2.5 months. About the follow of the last treatment 53 (46.5%) patients declared a rigorous treatment, 15 (13.1%) somewhat close and 46 (40.4%) patients loosely followed. All the patients declared that after the last treatment, the disease reappeared and spread again. They did not notice that medication taken for other health conditions affected vitiligo.

### 3.5 Skin condition

Regarding the presence of gray hair, 56 (49.2%) patients declared that they had, and 58 (50.8%) patients denied it. Regarding the family history of early hair graying 35 (30.7%) patients answered positively. To the question “Do you sunburn easily?” 69 (60.5%) of the patients answered yes, and 45 (39.5%) patients answered no. In response to the presence of a halo nevus (benign mole with depigmented ring or Sutton naevus) on the body frequently associated with vitiligo 33 (28.95%) patients answered yes, and 81 (71.05%) patients answered no.

### 3.6 Other conditions

Regarding the presence of a known allergy 10 (8.7%), patients declared that they had, from these 4 patients had a food allergy, 4 patients had a medication allergy, and 2 patients were allergic to insect stings. The presence of other autoimmune diseases associated with vitiligo is common. The following autoimmune diseases were detected in the studied group: 11 (9.7%) cases of psoriasis, 20 (17.5%) cases of diabetes, 30 (26.3%) cases of autoimmune thyroiditis, and 4 (3.5%) cases of rheumatoid arthritis. Regarding the presence of skin tumors in the studied group, only 3 (2.6%) patients declared the presence of non-melanoma skin cancer. The presence of the disease in the family is frequent. In the studied group, 34 (29.8%) of the patients were confirmed to have the disease in their first or second-degree relatives.

### 3.7 Impact

In examining the costs of the treatment, we have found no analyzable data.

## 4 Discussion

Demographic data: Vitiligo is the most commonly acquired disorder of pigmentation, with a prevalence between 0.5 and 2% of the world population on average. Some authors, like Bibeau et al. (14), report a prevalence of ≤ 0.6%. Prevalence rates vary geographically and are often higher in Africa and India. Vitiligo in dark-skinned populations and/or in regions, where social stigmas are important leads to a higher reported prevalence (15). Clinical studies have found the prevalence to be 0.91% in China (16), in India, the hospital-based point prevalence was 9.98% (17), compared to 0.38%, in Denmark (18). Gujarat in India is considered to have the highest prevalence in the world with 8.8% of the local people affected by vitiligo (19). Male and female are equally affected by vitiligo, most of the clinical studies confirm this data. Bibeau et al. (14) in a comprehensive study with 35,694 participants from three continents conclude an equal distribution of gender rate, with a higher presence of the disease in the younger group of ages and especially in females. The results are similar to our results regarding the gender rate and the presence of the disease in the younger group, but we had equal distribution between genders. The results of the studies conducted regarding the residence of the patients, and marital status have an important impact on psycho-social and health-related quality of life which is not the theme of this paper. The patients from the cohort were majority urban residents, with more than 95% of them with medium and high finished studies and 67.58% of them married. These factors can be important in accessing basic and specialized medical services. The last question from this subgroup was about the color of the eyes. The color of the eyes is linked to phenotype and genetics. People with phenotypes I to III, and especially Caucasians are predisposed to have light-colored eyes (20). Of the patients studied, 70 (61.4%) have light-colored eyes, like blue, gray, and green. The presence of light-colored eyes could be a predisposing factor in the appearance of vitiligo.

History of vitiligo: Regarding the age of the first signs of vitiligo, we can conclude that more than 55% of the patients belonged to group 3, with more than 10 years of appearance of the disease. About the first location of the disease, we note that in some people the first signs of the disease were distributed and appeared in more than one place. We have chosen the larger surfaced lesion as the first clinical sign. In more than 75.44% of the cases, the first clinical sign was on the visible area, head, hands, and foot region. As possible causes of the appearance of vitiligo, the patients declared in 71.93% of the cases that emotional stress could be a major factor. Silverberg and Silverberg (21) in a study found that 56.6% of participants had a stressful life event within the 2 years before vitiligo onset. Manolache et al. (22) in a case-controlled study found stressful events in 12 of 21 (57%) Romanian children with vitiligo, which was higher than controls. Pregnancy is a known trigger for vitiligo disease onset, but not all females with pre-existing disease experience exacerbation during pregnancy. In our cohort, 9,65% declared pregnancy as a causative factor. Regarding the influence of pregnancy on vitiligo, it was found that only 17.54% of the cases had worsening of the disease during pregnancy (23). Sunburn and trauma of the skin (Köbner phenomena) are well-known factors, that can start the illness. We found that in 18.42% of the cases, these two factors were present, being higher in comparison with database data (24). Redness or itch are clinical signs that can be present in vitiligo. Also, they can be present before the onset of the disease. Vachiramon et al. (25) in a study concluded that itch can be present in 20.2% of the cases. In our study regarding the question 82 (72%) patients answered yes and 32 (28%) answered no. We consider that the increased result may be a coincidence.

Vitiligo description: Skin type was determined by the skin photo-typing method proposed by Fitzpatrick. Our patients were all Caucasians belonging to photo type I–III, the majority (72%) of them were in phototype II. Vitiligo affects people of all races equally; the spots tend to be more noticeable in people with brown or Black skin (26). Ahn et al. (27) in a Korean population study found that skin type II was significantly less frequent and skin type V was quite common in the vitiligo group. These results suggest that people with dark skin have a higher probability of developing vitiligo than people with light skin (27). Regarding the clinical forms that we have grouped in visible and invisible areas containing 47 (41.2%) patients, respectively, 67 (58.8%) patients, we found similar results to Bibeau. The affected surface is a criterion for severity. Also we have found that most of the patients belonged to the minor and moderate form, like those patients in the study conducted by Bibeau. Vitiligo lesions in visible areas are associated with self-consciousness, and with a reduced quality of life (14).

Vitiligo treatments: Analyzing the results of the treatments used we conclude that more than 80% of the patients used treatments according to the protocols in force (28). More than 60 % used local treatment with potent steroids, calcipotriol, and their combination with, greater or lesser effectiveness. Also, about 20% of them had used phototherapy like PUVA, Narrow Band UVB, or excimer laser treatment. Other kinds of treatments like systemic, surgical, traditional, empiric, or complementary were also used in some cases. New treatment methods like CO_2_ laser, combined laser, low-dose cytokines-based, and topical or systemic anti-JAK drugs, are yet not available at the moment in our country (29–32). Regarding the evolution, the stability of the disease, and the follow-up of the treatment by the patients, the results are essentially similar to the data described in the literature by Marinho et al. (33), van Geel et al. (34), and de Barros et al. (35).

Skin condition: The appearance of gray hair is frequent in patients with vitiligo and their families (36). A large population-based study reported that 6–23% of people have 50% gray hair by 50 years of age (37). The presence of gray hair in patients with vitiligo probably is linked to common causes of developing the disease like stress, oxidative stress, autoimmunity, and low B vitamin levels (38, 39). This hypothesis is confirmed by the marked percentage of gray hair in our studied cohort and their families. Regarding the question Do you sunburn easily? patients answering with yes in 60.5% of the cases is probably due to the lighter phenotype of the cohort, these patients being predisposed to that. The presence of halo nevus (benign mole with a depigmented ring or Sutton naevus) on the body is frequently associated with vitiligo as mentioned in many studies as being a risk factor for developing the disease or a marker for evolution (40). Patrizi et al. (41) in a study mention that in patients with multiple halo naevus, the risk of vitiligo and other autoimmune diseases seems to be higher than in patients with single halo naevus. Zhou et al. (42) in a study of a total of 212 patients with halo naevus, from which 101 patients had also vitiligo suggests that earlier onset age of halo naevus might be associated with a higher risk of developing vitiligo. van Geel et al. (43) analyzing 291 patients with halo naevi divided into three groups as follow: patients with only halo naevi (group 1; *n* = 40), patients with generalized vitiligo without halo naevi (group 2; *n* = 173) and patients with generalized vitiligo with halo naevi (group 3; *n* = 78), concluded the hypothesis that halo naevi can represent a distinct condition, and may be an initiating factor in the pathogenesis of vitiligo. The high presence of 28.95% of the patients with the presence of halo naevus in our cohort, seems to support these hypotheses.

*Other conditions:* The presence of self-reported different types of allergies has a range between 9 and 35% of the population worldwide. Our results are much lower than we have found in the literature (44, 45). The presence of other autoimmune and systemic diseases associated with vitiligo is frequent. *Oguz Topal et al.* (46) in a study found that 45% of the patients had an associated systemic disease. Autoimmune thyroid disease, essential hypertension, and alopecia areata, which were observed in 28, 8, and 5% of patients, respectively, were the most common associated diseases. *Sheth et al.* (47) mentioned that 23% of the 3,280 studied patients had one of the following comorbidities: 287 thyroid-related, 186 psoriasis, 72 rheumatoid arthritis, 59 alopecia areata, 55 inflammatory bowel disease, 53 systemic lupus and 20 type I diabetes mellitus. The most frequent autoimmune disease found as a comorbidity in vitiligo was autoimmune thyroiditis. *Gill et al.* (48) on 1,098 patients with vitiligo, concluded that nearly 20% had at least 1 co-morbid autoimmune disease. Compared the results with the general US population, they found a higher prevalence of thyroid disease *(48)*. Another researcher, *Gey et al.* (49) found, that of 626 patients studied with vitiligo, 131 suffered from thyroid disease and recommended monitoring them for thyroid function. *Dahir and Thomsen* (50) found similar results in their study. Our results confirm the high presence of thyroid disease among other autoimmune diseases in vitiligo. Regarding the presence of skin tumors in the studied group, only 3 (2.6%) patients declared the presence of non-melanoma skin cancer whose incidence is much lower than in the general population in our country *(51)*. The appearance of vitiligo in the first or second-degree relatives is frequent. The majority of vitiligo cases are sporadic, but the familial presence is not uncommon, and generally, up to 20% of patients report the presence of the disease in the family. The frequency of vitiligo among first-degree relatives in Caucasians, Indo-Pakistani, and Hispanic populations is 7.1, 6.1, and 4.8%, respectively, compared to an estimated worldwide frequency of 0.14 to 2% *(52)*. Phiske (53), in a study, presents that up to 12 to 35% of pediatric vitiligo patients have family members with the disease. *Wang et al.* (24) in a questionnaire-based study on 17,345 patients found a prevalence of vitiligo of 0.56%, with a positive family history of 9.8% of them. In our studied group, 34 (29.8%) of the patients were confirmed to have the disease in the family, which is higher than the results from other studies.

## 5 Conclusion

In our study, we found that most of the clinical and epidemiological characteristics of vitiligo in our patients were similar to those in other studies. Some of the characteristics linked to the possible appearance of the disease were present in higher percentages like the presence of the disease in the family, lighter color of the eyes, gray colored hair, the presence of the halo naevus, the predisposition to sunburn, the skin trauma as the starting cause and the presence of increased level of thyroid disease. Based on our results, we can conclude a profile of a potential patient who can develop vitiligo and who would present the following characteristics: male or female with phenotype I–III, with light-colored eyes, showing halo naevus, having a personal history of multiple traumas of the skin, presenting gray hair itself or also in the family, who often burns in the sun, has the disease in the family and suffers from thyroid damage or other autoimmune diseases. To our knowledge, this study is the first of its kind from our country, however, our inferences remain limited by the single center, a relatively small sample size, recall bias, and a self-decided classification of some clinical aspects, which are potential limitations of this study.

## Data Availability

The original contributions presented in the study are included in the article, further inquiries can be directed to the corresponding author.
